# Platelet-rich plasma as an adjuvant therapy to fractional ablative carbon dioxide lasers for cutaneous repair: a complementary treatment for atrophic acne scarring

**DOI:** 10.1007/s10103-024-04192-y

**Published:** 2024-10-10

**Authors:** Simonetta I. Gaumond, Rama Abdin, Marita Yaghi, Rami H. Mahmoud, Mario Rodriguez, Ariel E. Eber, Joaquin J. Jimenez

**Affiliations:** 1 Dr. Phillip Frost Department of Dermatology and Cutaneous Surgery, University of Miami Miller School of Medicine, Miami, USA; 2 Department of Biochemistry and Molecular Biology, University of Miami Miller School of Medicine, Miami, USA; 3 Charles E. Schmidt College of Medicine, Florida Atlantic University, Boca Raton, USA

**Keywords:** PRP, Laser, Fractional laser, Resurfacing, Scarring, Acne

## Abstract

Acne has a prevalence of over 90% among adolescents, and subsequently progresses to acne scarring in approximately 47% of cases. Due to the severe psychological and social ramifications acne scarring has on patients, there is a need for more effective treatments. Platelet-rich plasma (PRP), an autologous preparation enriched with growth factors, cytokines, and chemokines, has shown efficacy in promoting wound healing and tissue remodeling in dermatology. Recent evidence suggests that the efficacy of PRP may be enhanced when combined with laser therapy, which induces controlled tissue damage through photo-thermolysis thereby promoting tissue remodeling and epidermal regeneration. The microchannels created by laser treatments are thought to allow deeper penetration of PRP into the skin, potentially increasing its therapeutic effects. This review aims to analyze the combined use of PRP and laser therapy for treating acne scarring by examining randomized control trials from the past decade indexed on PubMed. Six studies met our inclusion criteria and were included in this review. The findings of this review support the hypothesis that combining PRP with laser therapy offers superior clinical results compared to monotherapy, providing a more effective approach to managing acne scarring.

## Introduction

### Acne scarring: epidemiology, pathophysiology, and classification

Acne vulgaris is one of the most common skin afflictions worldwide, having a prevalence of over 90% among adolescents [1]. Recently, there has been a notable increase in the persistence of acne into adulthood, with specific predispositions linked to gender, familial history, and geographical factors. A comprehensive meta-analysis of 37 studies by *Liu et al.* [2] demonstrated that 47% of individuals with acne vulgaris subsequently develop acne scarring. The prevalence of acne scars also exhibits geographical variation, with higher prevalence in Asia (52%), Europe (51%), and North America (50%), compared to Africa (31%) and South America (20%) [2]. Importantly, significant psychological ramifications are attributable to acne scarring, including a decreased quality of life, increased rates of depression, and an increased risk of suicide [3]. Therefore, it remains imperative to find more effective treatments targeting atrophic acne scarring and mitigating their psychosocial burden.

Superficial cutaneous injuries typically heals without scarring, however, trauma to the dermis leads to visible scar formation [4]. Atrophic scars are characterized by aberrant production and degradation of collagen during the healing process [5]. In approximately 85% of cases of acne scarring, there is a net destruction of collagen in the dermis resulting in atrophic scars [6]. In the other 15% of cases, a net gain of collagen results in atypical scars of hypertrophic or keloid morphology [6]. Scar formation is part of the wound healing process, which is divided into 3 distinct phases: inflammation, granulation tissue formation, and remodeling [7]. The inflammatory phase typically lasts less than 72 h and involves hemostasis, chemotaxis, and increased vascular permeability. These steps limit further damage by clearing debris and bacteria and closing the wound [7]. This phase is characterized by the skin being visibly swollen and erythematous due to the dilation of blood vessels [8]. Next, the granulation tissue formation stage begins and lasts up to 6 weeks [7]. In the early stages, stimulation of cytokines such as platelet-derived growth factor (PDGF), epidermal growth factor (EGF), transforming growth factor β (TGF-β), and fibroblast growth factor (FGF) by macrophages increases the number of fibroblasts [8]. Stimulation of TGF-β1 and TGF-β2, specifically, controls the downstream formation of scar tissue [9]. Subsequently, the process of granulation tissue formation, which contains a network of capillaries, fibroblasts, and white blood cells, combined with the synthesis of collagen production begins [8]. Together, granulation tissue and collagen fill the skin defects [8]. First, type III collagen is produced, which is then replaced by the more elastic type I collagen [8, 9]. Dysregulation in the production and degradation of collagen during this phase of the wound healing process is what leads to the development of atrophic acne scars [4, 10]. In the final remodeling phase, rebuilding and maturation of the scar transpires which can last up to 12 months [7]. The granulation tissue is replaced by fibrous tissue which contracts the wound [8]. Contraction of scar tissue develops into small soft tissue defects resulting in an atrophic scar morphology on the surface of the skin [4]. Because atrophic acne scars form deeper than surface wounds, acne scarring treatments must target deeper cutaneous tissue to achieve clinical efficacy.

*Jacob et al.*. divided atrophic acne scars into three general categories: icepick, boxcar, and rolling scars [11]. These classifications are based on the width, depth, and three-dimensional structure of the collagen degradation [11]. Ice pick scars are V-shaped, smaller than 2 mm in diameter, and extend vertically to the deep dermis or subcutaneous tissue [6, 11]. Boxcar scars are wider scars that range from 1.5 to 4 mm in width and are characterized as depressions with sharply demarcated vertical edges [11]. Finally, rolling scars are the widest, with a range between 4 and 5 mm in diameter, and provide an undulating appearance to the skin [5, 6, 11]. Since it has been reported that specific treatments yield more optimal results for specific types of scars, such as lasers for boxcar scars, an analysis of acne scarring subtype should be conducted to tailor the treatment method to the predominant scar type present [6, 10]. A patient-specific treatment plan will maximize cosmetic results and likely yield the highest patient satisfaction.

## PRP and laser mechanism of action

### PRP

Platelet-rich plasma (PRP) is an autologous preparation consisting of a high concentration of human platelets in a small plasma volume. There are many methods used to obtain PRP. The most common way of obtaining PRP is to withdraw blood from the patient, collect it in a sterile tube containing an anti-coagulant, and perform a double centrifugation technique [12, 13]. To achieve platelet degranulation and release of bioactive molecules, PRP is typically activated prior to use with an aggregation inducer, such as calcium chloride [14]. The growth factors released from the alpha-granules of concentrated platelets include, PDGF, TGF-β1 and 2, EGF, vascular endothelial growth factor (VEGF), and insulin-like growth factor (IGF) [15, 16]. These growth factors are responsible for chemotaxis, cell proliferation and differentiation, and angiogenesis [15]. PRP also contains cytokines, chemokines, and other bioactive molecules, such as IL-8 and endostatins [15]. These stimulate tissue remodeling by attracting macrophages, regulating angiogenesis, upregulating collagen and protein synthesis, promoting cellular proliferation, and increasing fibroblast production [17]. In fact, there is histological evidence that the injection of PRP in the deep dermis can induce soft-tissue augmentation, activate fibroblasts and new collagen deposition, and stimulate new blood vessel and adipose tissue formation [18]. Another essential component of PRP are proteins, such as fibrin, fibronectin, and vitronectin, which promote cell adhesion [15]. They provide the structural support necessary for cell migration, proliferation, and the 3-dimensional growth of tissues over these structures [15]. Therefore, PRP not only stimulates the release of growth factors, but it also exerts a wider influence on the extracellular matrix, aiding in tissue repair and regeneration. Due to its autologous nature, PRP is considered a safe treatment that by definition lacks the potential risks of disease transmission associated with using blood products from donors. To date, no systemic side effects have been reported related to the growth factors released from administration of PRP [15].

### Laser

Lasers are very effective in treating acne scars, especially in patients presenting with boxcar scars or rolling scars [6]. They work by creating short-pulsed light energy in a defined pattern [5]. The photo-thermolysis produces non-continuous columns of tissue destruction in the dermis and epidermis [6, 10]. These micro-channels promote epidermal regeneration by triggering tissue remodeling and synthesis of new collagen and elastic fibers [6, 10]. In the setting of fractional ablative lasers, the tissue surrounding each column is spared, allowing for faster re-epithelization and optimal wound healing [6]. Fractional carbon dioxide (FCL) lasers, specifically, emit a peak wavelength of 10,600 nm, which is absorbed by the epidermis, targeting the most superficial cutaneous layers and promoting re-epithelization [19]. The thermal injury created by this laser causes heat-meditated contraction of collagen fibers, thereby stimulating collagen remodeling and skin tightening. Compared to other lasers, CO_2_ lasers produce more thermal energy and increase coagulation of small blood vessels in the dermis, resulting in a significant bleeding reduction when treating larger surfaces [19]. Side effects associated with lasers include infections, procedural discomfort, persistent erythema, hyperpigmentation, and scarring [5].

### Combination

It has been suggested that combining PRP with laser therapy imparts synergetic effects for acne scarring [20]. The microchannels produced by the laser therapy enable PRP to penetrate deeper within the skin, allowing for enhanced efficacy and better clinical outcomes [20]. With more significant product penetration, the growth factors and cytokines from PRP are more optimally absorbed and therefore have more potent effects [20]. The cytokines secreted by PRP may also reduce post-treatment erythema [20, 21] and alleviate discomfort associated with laser treatment [22, 23]. Altogether, autologous PRP is hypothesized to enhance the effects of laser treatments resulting in clinically superior tissue remodeling with fewer adverse effects.

## Methods

A review of existing studies indexed as “randomized control trials” on PubMed over the last decade was performed, which evaluated PRP as an adjuvant treatment to lasers for the treatment of acne scarring. The literature search was conducted using key words such as “platelet-rich plasma,” “lasers,” and “acne scarring”. Six studies evaluated the efficacy of PRP in combination with fractional carbon dioxide (CO_2_) laser for the treatment of acne scarring over the past 10 years and were included in this review. Manuscripts were excluded if they were not written in English, not peer-reviewed, or tested on non-human subjects. Reviews, case reports, case series, and commentaries were also excluded from this review.

## Results

From the six randomized control trials included in this literature review, two originated in South Korea [20, 24] and four in Egypt [21–23, 25]. A total of 189 patients within the age range of 19–44 were investigated, of which the mean age was 27 years. Although one study [20] did not mention the sex of the participants, the remaining 164 subjects examined included 67 males and 97 females. The exclusion criteria among these randomized control trials included patients with a history of hypertrophic scars or keloid formation, isotretinoin use within the last 6 months, diabetes, use of immunosuppressive medication or photosensitive drugs, collagen or vascular disease, active skin infection, lasers or chemical peels within the last 6–12 months, and patients with bleeding disorders. Additionally, pregnant or lactating women were also excluded from these studies.

These studies also varied in the methods of preparation and administration of PRP. One study [24] opted for the use of a commercially available kit (Prosys PRP, T Cell Bio Inc., Seoul, South Korea) while the other studies prepared the PRP manually. Typically, 10mL of blood was withdrawn from the subjects in a syringe pre-filled with an anti-coagulant at a 10:1 ratio. The most frequent anti-coagulant used was sodium citrate [21, 22, 25], although one study opted for acid citrate dextrose [23], and two studies did not specify [20, 24]. The method of centrifugation as well as the centrifugation speed and duration varied greatly between studies. Finally, three studies opted to activate the PRP prior to application, using either 3% calcium chloride [23], 10% calcium chloride [22], or 10% calcium gluconate [25]. One study did not specify which activation agent was used [20], and two studies did not activate the PRP prior to application [21, 24]. The results from these studies are shown in Table 1.

**Table 1:** Literature summary of the RCTs investigating the efficacy of combination FCL-PRP treatment for acne scars

Study	Sample size	FSP	Acne Scar Severity	Intervention	FCL parameters	PRP parameters	Study parameters	Results
*Lee et al.* [24]	14	III-V	Moderate-to-severe	**Split face** FCL + ID saline vs. FCL + ID PRP **Regimen**: 2 treatments at 1 M intervals **Last FU**: 4 M after last treatment	Pulse energy: 25 mJ per fixed 150 μm diameter microbeam Density: 400 MTZ/cm^2^ Cooling: concurrent forced air	0.3mL injections (20) spaced at 1.5–2 cm intervals	• Photography-based physician-assessed overall clinical improvement (4-point scale) • Erythema: 5-point scale and chromameter • Edema: 5-point scale • AEs assessed: petechiae, oozing, crusting, dyschromia, scarring	• Significantly better clinical improvement with ID PRP (2.7 ± 0.7 vs. control: 2.3 ± 0.5; *p* = 0.03) was observed 4 M after the final treatment • Lower intensity of physician-assessed erythema on ID PRP treated facial halves at day 4 (*p* = 0.01), also confirmed by chromameter (*p* = 0.049) • Duration of erythema shortened with ID PRP use (8.6 ± 2.0 days vs. control: 10.4 ± 2.7 days; *p* = 0.047) • Duration of edema shortened with ID PRP use (6.1 ± 1.1 days vs. control: 7.1 ± 1.5 days; *p* = 0.04) • Shorter duration of post-treatment crusting on the side treated with ID PRP (5.9 ± 1.1 days vs. control: 6.8 ± 1.0 days; *p* = 0.04) and no petechiae, oozing, dyschromia, infection, scarring, or blistering otherwise occurred in any participant.
*Gawdat et al.* [23]	30	III-V	Moderate-to-severe	**Split face** Group 1: FCL + ID saline vs. FCL + ID PRP Group 2: FCL + ID saline vs. FCL + topical PRP **Regimen**: 3 treatments at 1 M intervals **Last FU**: 3 M after last treatment	Power: 15 W Dwell time: 600μs Spacing: 700 μm Stack: level 2 Cooling: ice packs	0.2mL injections (10) spaced at 1.5 cm intervals	• Photography-based physician-assessed skin smoothness (4-point scale) • Patient clinical satisfaction • Acne scar depth: OCT imaging • Pain: 9-point scale • AEs assessed: erythema, edema, crusting, PIH, acneiform eruptions	• Clinical improvement as per physician’s assessment revealed improved skin smoothness significantly with FCL-PRP combination treatment as compared to control (*p* = 0.03) o ID PRP = 66.7% achieved excellent improvement o Topical PRP = 60% achieved excellent improvement o Controls = 26.7% achieved excellent improvement • Patient assessment comparable to physician assessed results • FCL-PRP combination treatment showed significantly greater improvement in acne scar depth as compared to control (*p* = 0.01), with no significant difference between ID and topical PRP • Pain levels were significantly greater in the areas treated with ID PRP as compared to topical PRP or control (mean: 7.2 (± 1.2) vs. 2.8 (± 0.6) vs. 3.0 (± 0.7), respectively; *p* = 0.005) • Post-FCL AEs were of shorter duration in the areas treated with ID or topical PRP as compared to control (*p* = 0.02)
*Min et al.* [20]	25	III-IV	Moderate-to-severe	**Split face** FCL + ID saline vs. FCL + ID PRP **Regimen**: 2 treatments at 1 M intervals **Last FU**: 2 M after last treatment	Fluence: 30-70 mJ/cm^2^ Density: 150 MTZ/cm^2^ Spot size: 12 mm Pulse duration: 1ms	0.02mL injections spaced at 1–1.5 cm intervals	• Investigator’s Global Assessment (IGA) (5-point scale) and ECCA • Skin recovery: epithelization scale • Erythema: spectrometry • AEs: Visual analogue scale (VAS) • Patient-reported satisfaction • Biopsy with IHC staining	• Significantly superior clinical improvement was reported with the use of ID PRP as per the IGA (75% improvement vs. 50% improvement for control; *p* < 0.001) and ECCA (*p* < 0.05) • Less erythema was consistently measured using both the erythema index and colorimetric measurements on the ID PRP-treated sides (*p* < 0.05) • As for AEs, all patients experienced initial erythema and PIH which were not resolved by last FU in four PRP-treated sides and five control sides. Additionally, only control sides showed increased amounts of swelling for > 7 days after each treatment session • Patient-reported satisfaction at the last FU was significantly higher with FCL-PRP combination therapy (*p* = 0.016) and correlated with physician-reported scores on the improvement scale • Focusing on the molecular findings, collagen-1 (*p* = 0.03) and collagen-3 (*p* = 0.03) expression was found to be significantly greater in the PRP-treated sides at day 28. Higher levels of TGFβ-1 (*p* = 0.03) and TGFβ-3 (*p* = 0.04) at day 28 were also found on the sides treated with PRP. In addition, the use of PRP was also associated with increased c-myc expression (*p* = 0.04)
*Galal et al.* [21]	30	IV-V	Mild-to-severe	**Split face** FCL + ID PRP vs. FCL only **Regimen**: 3 treatments at 1 M intervals **Last FU**: 1Y after last treatment	Power: 15 W Spacing: 800mμ Dwell time: 600sμ Stack: level 2	N/A	• Acne scar severity: qualitative & quantitative Goodman & Baron scales • Skin roughness, acne scar depth, erythema, and pigmentation: skin analysis camera system (Antera 3D™) • AEs assessed: erythema, edema, crust formation • Patient satisfaction: 3-point Likert scale	• FCL-PRP combination treatment led to significantly improved scar depth as per the Goodman and Baron scale as compared to control (*p* < 0.0001) • Sides treated with combination FCL-PRP harbored greater significant improvement in scar depth, erythema, and pigmentation as per the Antera scoring system (*p* < 0.001) o Treated with FCL-PRP combination: 70% showed good or excellent improvement o Control: 30% showed good or excellent • AEs resolved within 3 days on the side treated with PRP as compared to 5–7 days on FCL only treated sides • Additionally, 50% of participants reported satisfaction with FCL-PRP combination as compared to 3.3% with FCL treatment only
*Mahamoud et al.* [25]	30	III-V	Mild-to-severe	**Split face** FCL + ID PRP vs. FCL + HA **Regimen**: 3 treatments at 1 M intervals **Last FU**: 3 M after last treatment	Power: 15–20 W Spacing: 800–1000 μm Dwell time: 500-800ms Stack: level 2	*PRP*: 0.2-0.3mL injections *HA*: 16 mg/mL Viscoderm™	• Photography-based physician-assessed overall clinical improvement (4-point scale) • Physician global assessment: qualitative and quantitative Goodman & Baron scales • Pain: 0–10 numeric rating scale (NRS) • AEs assessed: downtime, erythema, edema, PIH, pain, scarring, milia, infections • Skin punch biopsies for H&E, Masson trichrome, and orcein staining • Patient satisfaction score (4-point scale)	• Improvement was more pronounced on quantitative Goodman and Baron assessment with FCL-PRP (33%) as compared to FCL-HA (21.72%) (*p* < 0.001) • As per the qualitative Goodman and Baron scale, post-treatment improvement in scar severity was similar with both modalities (*p* = 0.921) • Pain NRS of 5.57 ± 1.81 on the PRP treated side vs. 6.00 ± 1.98 on the HA treated side • Post-procedural AE duration was similar considering overall downtime, edema, and erythema • Improvement in mean epidermal thickness (FCL-PRP: 67.7% vs. FCL-HA: 52.8%), collagen mean area percent (FCL-PRP: 86.3% vs. FCL-HA: 56.5%), and elastin mean area percent (FCL-PRP: 68.0% vs. FCL-HA: 46.1%) following treatment with both modalities • Patients’ satisfaction scores and mean pain levels (FCL-PRP: 5.57 ± 1.81 vs. FCL-HA: 6.00 ± 1.98) failed to demonstrate superiority of either modality
*El-Hawary et al.* [22]	60	III-V	Mild-to-severe	**Multi-arm** Group 1: ID PRP Group 2: FCL Group 3: FCL + ID PRP **Regimen**: 3 treatments at 1 M intervals **Last FU**: 6 M	Power: 12 W Dwell time: 600ms Spacing: 600ms Stack: level 2	0.1mL injections spaced at 1 cm intervals	• Photography-based physician-assessed overall clinical improvement (4-point scale) • Qualitative Goodman and Baron scale • Participant satisfaction: 4-point scale • Pain: 5-point scale • Patient-assessed AEs (5-point scale) • Skin punch biopsies for H&E, Mallory trichrome, and Verhoeff Van Gieson staining	• Treatment with either FCL alone or FCL-PRP led to significantly better improvement than with PRP alone (*p* = 0.028 and *p* = 0.002, respectively) but failed to demonstrate the superiority of either modality when comparing group 2 to group 3 (*p* = 0.657). • Patient satisfaction for scar appearance, skin texture, and overall satisfaction was statistically significant and higher in group 2 and 3 than in group 1 • Pain was more severe with FCL-PRP combination treatment, but erythema, edema, and crust formation were more severe and lasted longer with FCL treatment alone. Similarly, downtime was longer with FCL treatment alone than with combination FCL-PRP (FCL alone: 5.40 ± 1.07 days vs. FCL-PRP: 4.60 ± 0.84 days) • A significant increase in epidermal thickness, percentage of collagen fibers, and Ki-67 expression, reflecting an increased number of proliferating epidermal cells, across all treatment groups but the percentage of elastic fibers was only increased with the use of either FCL alone or combination FCL-PRP, the latter showing a greater increase

## Discussion

Laser therapy is a promising therapeutic option for the treatment of acne scarring, especially when combined with other therapeutic modalities such as PRP. For the purpose of this review, six studies [20–25] investigating combined ablative fractional CO_2_ laser and PRP treatments were analyzed, and all studies offer support for the efficacy of combination therapy. It is important to note that studies varied in laser therapy protocols, including pulse energy, pulse density, and frequency of laser treatments. While the clinical implications of these variations are not completely understood, they are worth mentioning, and can be found in Table 1. A recent international panel, comprised of 24 dermatologists and plastic surgeons, released consensus recommendations regarding the role and efficacy of energy-based devices for treating acne scarring [26]. Important considerations include the patient’s skin type, acne scar type, scar severity, and pain tolerance [26]. The panelists almost universally (91%) agreed that superficial boxcar scars were the most likely type of acne scar to respond to energy-based treatment, while icepick scars were classified as the least likely type to respond to therapy [26]. In our review, one study found better improvement in rolling and ice pick scars than boxcar scars, which is not in concordance with panel findings [21]. This emphasizes the relevance of studies reporting patient scar type in future studies to clarify and contextualize laser therapy efficacy in treating different acne scar types. Furthermore, regarding general assessment of scar severity, the studies varied in their qualitative and/or quantitative scoring systems which introduces an additional obstacle in the objective comparison of study findings.

All studies investigated intradermal PRP use [20–25], and one additionally evaluated topical PRP treatment [23]. The method of PRP administration can be influential since there is a potential for more efficient product penetration, leading to additional wound healing and regeneration with ID treatment [24]. This theory begets questions on the comparison of topical and intradermal PRP in combination with FCL therapy. *Gawdat et al.* found that although combination topical or intradermal PRP with FCL therapy is superior to FCL alone, there was no significant difference in efficacy between intradermal and topical PRP combination groups [23]. This may be due to enhanced topical PRP delivery with laser therapy through microchannels, and suggests that topical PRP may be a more favorable adjunct to laser therapy as it is associated with less pain than intradermal PRP injection [23].

For clinicians, important considerations when discussing laser therapy as a treatment option for patients with acne scarring are the patients’ pain tolerance, skin type, and available post-procedure downtime as these all relate to potential laser therapy adverse effects. These include erythema, edema, dermatitis, acneiform reaction, herpes simplex virus and varicella reactivation, and dyschromia [26]. Hyperpigmentation is a known complication of energy-based treatments, particularly in patients with darker skin (e.g. Fitzpatrick skin type IV-VI) [26]. In this review, Fitzpatrick skin types ranged from III-V. Hyperpigmentation is due to dysregulation of the basal cell layer and increased melanogenic activity of epidermal melanocytes [23, 27]. Although hyperpigmentation was noted to be a side effect in some studies [20, 22, 23], it was less prevalent, or even non-existent, in groups treated with combination therapy. *Gawdat et al.* propose that TGF-β secreted by PRP may decrease melanogenesis and enhance production of basement membrane proteins which expedite basement membrane repair, thus thwarting dyschromia [23]. These studies show promising results for patients with darker skin phototypes, however, patients should always be warned against the potential for hyperpigmentation with laser treatment, even when combined with PRP therapy. This adverse effect can be mitigated through decreased fluence, reduced pulse density, longer time intervals between treatments, longer pulse durations, cooling methods, topical therapy such as corticosteroids, and sun protection [26].

Combination therapy was also associated with reduced post-procedure erythema and edema [20, 21]. This may be due to growth factors within PRP, such as PDGF and TGF-β, enhancing tissue remodeling and recovery [21]. Patients treated with FCL followed by intradermal PRP typically reported increased pain compared to topical PRP or FCL alone likely due to further trauma to skin which has already been sensitized by prior FCL therapy [22, 23]. It has also been suggested that topical PRP may decrease burning sensation caused by FCL therapy by bolstering the activity of dermal cells to create a protective barrier within the tissue that minimizes the transmission of pain downward from the surface of the skin [28]. Overall, it appears that combination therapy may not only enhance clinical efficacy but may also decrease post-procedure downtime and duration of some adverse effects, namely hyperpigmentation, erythema, and edema.

Histopathologic support for combination therapy includes increased epidermal thickness, percentage of collagen fibers, and percentage of elastic fibers when compared to FCL or PRP monotherapy [22, 25]. This is relevant given the pathologic mechanism of atrophic scarring which involves degradation and disequilibrium of collagen fibers [10]. These results are in concordance with those of *Min et al.* which reported significantly increased collagen types I and III in the combination treatment groups [20]. This enhanced efficacy with PRP is likely due to the secreted growth factors and cytokines which foster matrix formation, collagen production, and cellular multiplication [14]. Combination PRP and FCL treated groups were found to have significantly higher levels of TGF-β, which is known to promote collagen deposition and increased fibroblast proliferation, both of which translate into decreased scarring response [29–31]. In addition, the use of PRP was associated with increased c-myc expression, which is hypothesized to work synergically with TGF-β to augment epithelial–mesenchymal transitioning in wound healing [32]. This seems to suggest that both TGF-β and c-myc may play a role in the increase in collagen deposition, and therefore notable clinical improvement of acne scars seen with PRP treatment.

## Limitations

A limitation identified among the studies reviewed was the variance in the preparation and administration protocols of PRP between the studies (Table 1). There is no standardized protocol for PRP production as there is potential for variation in all phases of preparation including but not limited to centrifugation, anticoagulation, and activation [33]. These variables may affect PRP potency and efficacy, therefore any study using PRP should provide a detailed description of the preparation and administration protocols [34]. A recent scoping review of comparative studies investigating PRP preparation, storage, and or/administration uncovered the dramatic variabilities in PRP formulations, across several medical fields which utilize the product [33]. In this review, all [20, 22–25] except one study [21] opted for double centrifugation. Furthermore, the commercial kits used for PRP preparation also affect product properties such as platelet concentration [33, 35]. This is an important consideration as the optimal platelet concentration for PRP is not clear, and over saturation of platelets may actually impair wound healing [35]. Of the studies analyzed in this review, only one [25] reported platelet concentration within the prepared PRP. The optimal white blood cell (WBC) concentration for PRP is also unknown; similarly to platelet concentration, high concentrations of WBC may have pro-inflammatory and catabolic effects not conducive to wound healing [35]. Given that there is no consensus on the optimal regimen of PRP preparation and administration, studies using PRP should report key details such as baseline whole blood platelet count, centrifugal force, centrifugal time, PRP activation, concentration of platelets, WBCs, and growth factors.

Another limitation of this review is the search strategy utilized. The “randomized controlled trials” filter of PubMed was utilized to collect relevant studies, however, not all studies indexed as randomized controlled trials were as such. For example, *Mahamoud et al.* did not provide a reliable control group in their clinical trial comparing HA and PRP combination with laser therapy [25]. However, we proceeded with the investigation of all relevant trials populated during the search given the lack of randomized controlled trials to date, as well as the potential for the trials included which were not true randomized controlled studies to provide valuable therapeutic information. We hope to call attention to this shortcoming within the field and emphasize the need for future large scale randomized controlled trials investigating the combined use of laser and PRP given its demonstrated utility in preliminary clinical trials.

## Data Availability

No datasets were generated or analysed during the current study.
